# Geometric diffusion of quantum trajectories

**DOI:** 10.1038/srep12109

**Published:** 2015-07-16

**Authors:** Fan Yang, Ren-Bao Liu

**Affiliations:** 1 Department of Physics, The Chinese University of Hong Kong, Shatin, N.T., Hong Kong, China; 2 Centre for Quantum Coherence, The Chinese University of Hong Kong, Shatin, N.T., Hong Kong, China; 3 Institute of Theoretical Physics, The Chinese University of Hong Kong, Shatin, N.T., Hong Kong, China

## Abstract

A quantum object can acquire a geometric phase (such as Berry phases and Aharonov–Bohm phases) when evolving along a path in a parameter space with non-trivial gauge structures. Inherent to quantum evolutions of wavepackets, quantum diffusion occurs along quantum trajectories. Here we show that quantum diffusion can also be geometric as characterized by the imaginary part of a geometric phase. The geometric quantum diffusion results from interference between different instantaneous eigenstate pathways which have different geometric phases during the adiabatic evolution. As a specific example, we study the quantum trajectories of optically excited electron-hole pairs in time-reversal symmetric insulators, driven by an elliptically polarized terahertz field. The imaginary geometric phase manifests itself as elliptical polarization in the terahertz sideband generation. The geometric quantum diffusion adds a new dimension to geometric phases and may have applications in many fields of physics, e.g., transport in topological insulators and novel electro-optical effects.

When a discrete quantum eigenstate is adiabatically driven in the parameterized state space, in addition to the familiar dynamical phase, the state can acquire a geometric phase which depends on the gauge structure of the quantum system. In particular, the geometric phase accumulated along a cyclic evolution is the famous Berry phase, which is gauge invariant^1^,^2^. The geometric phase has played important roles in many fields of physics, as demonstrated by, e.g., the Aharonov-Bohm effect^3^, quantum Hall effect^4^,^5^,^6^,^7^,^8^, anomalous Hall effect^8^,^9^,^10^,^11^,^12^ and topological insulators^13^,^14^.

In contrast to a discrete eigenstate, a wavepacket in a continuum is a superposition of infinitely many eigenstates. For example, an electron wavepacket in an energy band is a superposition of the Bloch states. When the wavepacket is driven adiabatically along a trajectory in the parameter space, it can pick up a geometric phase similar to a discrete state^8^,^15^,^16^. Such geometric phases associated with electron wavepackets have important effects on electronic transports^8^. In addition, however, the wavepacket during the evolution will experience quantum diffusion (or dephasing) due to interference between different phase factors associated with different energy eigenstates that form the wavepacket. Then an interesting question arises: Does the quantum diffusion also have a geometric part?.

In this paper, we show that the geometric phase of a wavepacket along a quantum trajectory can have both real and imaginary parts. The imaginary part characterizes the quantum diffusion that is determined by the geometry of the quantum trajectory. As an example, we study the geometric phase of quantum trajectories in high-order THz sideband generation (HSG)^17^,^18^,^19^,^20^,^21^ in time-reversal symmetric semiconductors. We find that while the real part of the geometric phase leads to a Faraday rotation (FR) of the THz sideband emission, the imaginary part (i.e. the geometric quantum diffusion) manifests itself as the polarization ellipticity (PE) of the sideband. This finding extends the concept of the geometric phase to the complex plane, which may lead to a wealth of new physics. It may also have many applications in transport and electro-optics of novel materials such as topological insulators^13^,^14^.

## Results

### Imaginary geometric phase

**Figure 1:**
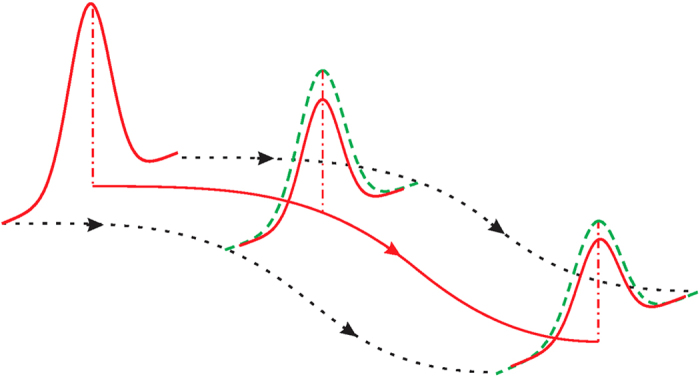
Schematics of the quantum trajectories of a wavepacket in the parameter space. The green dashed (red solid) Gaussian curves represent the diffusion of the wavepacket without (with) the geometric diffusion included. The red solid arrow represents the quantum trajectory that satisfies the stationary phase condition.

We consider a physical system described by a Hamiltonian *H*(**R**) that depends on time through the parameter **R**(*t*) = (*R*_1_(*t*),*R*_2_(*t*),…) (**R** will be the relative momentum **k** of an electron-hole pair for the specific example considered in this paper). Then the path integral form of the adiabatic evolution of a wavepacket is given by the propagator (see Fig. 1)
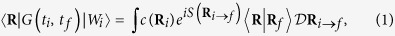
where 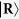 denotes an instantaneous eigenstate of *H*(**R)**, 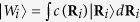 is the initial wavepacket at time *t*_*i*_, *G* is the propagator from *t*_*i*_ to *t*_*f*_, **R**_*i*→*f*_ denotes a path from **R**_*i*_ to **R**_*f*_, and *S*(**R**_*i*→*f*_) gives the action (i.e., phase) of the evolution along this path. In adiabatic evolution, the phase *S*(**R**_*i*→*f*_) (which acts as an action) can be decomposed into the dynamical phase *S*_*D*_ and the geometric phase *S*_*G*_, i.e., *S*(**R**_*i*→*f*_)=*S*_*D*_(**R**_*i*→*f*_)+*S*_*G*_(**R**_*i*→*f*_)^1^. In equation (1), the instantaneous eigen states with different parameters **R**_*i*_ will follow different paths **R**_*i*→*f*_ in the parameter space, and hence obtain different phases *S*(**R**_*i*→*f*_). The interference between these paths with different phases leads to quantum diffusion of the wavepacket. In this paper, we will identify the geometric quantum diffusion, which results from the interference between paths with different geometric phases.

To explore the geometric phase effects in the quantum interference, it is helpful to consider the semiclassical approximation, in which the summation of the phase factors along all possible paths is dominated by the orbits that satisfy the stationary phase condition *δS*[**R**_*cl*_] = 0, called quantum trajectories^22^,^23^ (which, in the standard path-integral formulation, are more often called semiclassical trajectories). Then the propagator is determined by the semiclassical actions of the quantum trajectories plus fluctuations nearby, that is,
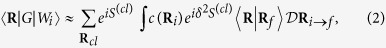
where
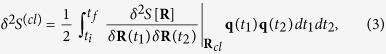
gives the quantum fluctuation around the quantum trajectories with **q** = **R** − **R**_*cl*_. Note that *S*^(*cl*)^ = *S*(**R**_*cl*_) = *S*_*G*_[**R**_*cl*_] + *S*_*D*_[**R**_*cl*_], where *S*_*D*_ is the dynamical part of the action and *S*_*G*_ is the geometric part, which depends only on the geometry of the path **R**_*cl*_ in the parameter space.

In the general case (e.g., in quantum tunnelling^24^), there may be no real orbit satisfying the classical equation of motion. Then we have to invoke analytic continuation of the classical mechanics to the complex plane. As a result, the geometric phase of the quantum trajectory also becomes complex with a nonzero imaginary part. Since the imaginary part of the action ℑ*S*^(*cl*)^ describes the quantum diffusion of the wavepacket due to the quantum interference, ℑ*S*_*G*_[**R**_*cl*_] represents the geometric part of the quantum diffusion. This geometric quantum diffusion results from the interference of the different geometric phase factors associated with a bunch of paths (i.e., the quantum fluctuations) near the quantum trajectory. Note that the imaginary part of the action causes a wavepacket decay, which signals the quantum diffusion. For example, the wavepacket decay in a quantum tunnelling can be regarded as quantum diffusion phenomenon. A main purpose of this paper is to quantify the quantum diffusion due to the geometric part of the imaginary action.

### Geometric phases in high-order THz sideband generation

**Figure 2:**
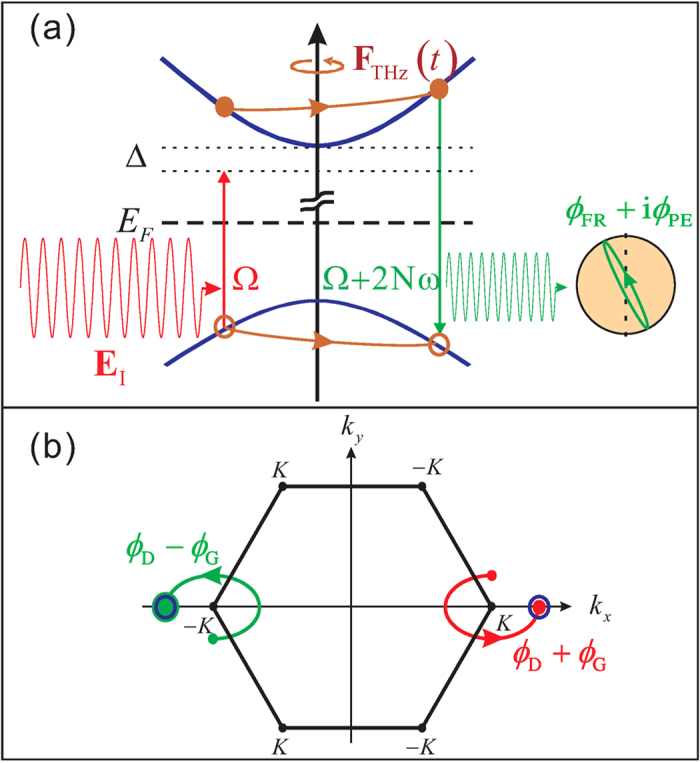
Schematics of high-order THz sideband generation and quantum trajectories in monolayer MoS2. (**a**) An electron-hole pair excited by a linearly polarized optical laser 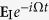 is driven along an elliptical quantum trajectory by the THz field, acquiring a kinetic energy, and recombines with emission of a sideband photon at frequency Ω + 2*Nω*. The sideband has a polarization ellipticity *ϕ*_*PE*_ and Faraday rotation *ϕ*_*FR*_, given by the imaginary and real parts of the geometric phase, respectively. (**b**) Interference of the quantum trajectories that contribute to the *N*th order sideband. The blue open circles represent the wavepackets at ±*K* valleys with dynamical diffusion only, while the filled circles are the wavepackets including the geometric diffusion.

To give a specific example of the geometric quantum diffusion, we consider the quantum trajectories of an optically excited electron-hole pair, driven by a terahertz (THz) field in a semiconductor^17^. The electron-hole pair, after excitation by a weak optical laser of frequency Ω, is driven into oscillations by an intense THz laser of frequency *ω*. The electron-hole pair subsequently acquires a kinetic energy, and recombines at a later time to emit photons at sideband frequencies Ω + 2*Nω*, with *N* being integers [Fig. 2(a)]. This so-called high-order THz sideband generation has been theoretically studied^17^ and recently experimentally observed^18^,^19^. In HSG the electron-hole wavepacket evolution is well approximated by a small number of quantum trajectories plus the quantum fluctuations around them. When the THz field is elliptically polarized, the quantum trajectories become curved in the momentum space. Our previous study shows that geometric phases will be accumulated along these trajectories if the energy bands of the semiconductors (such as monolayer MoS_2_ and bilayer graphene) have non-vanishing Berry curvatures^20^,^21^. The geometric phases result in an observable effect in time-reversal symmetric materials: The optical emission at integer multiples of the THz period after the excitation by a linearly polarized laser pulse has a Faraday rotation equal to the Berry phase accumulated along the quantum trajectory^20^,^21^. Therefore HSG in such materials provides an ideal platform for studying the geometric quantum diffusion (i.e., the imaginary geometric phase).

A general elliptically polarized THz field can be written as
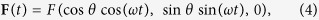
where *F* is the field strength. Under this field, the electron and hole will evolve along an elliptic path in the **k**-space,
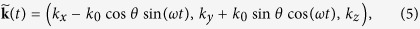
where *k*_0_ = *eF*/*ω* characterizes the maximum momentum gained from the THz field. The driving by the THz field is adiabatic in the sense that the THz field has a frequency much lower than the band gap of the material and hence induces no interband transition for the THz field strengths (~kV/cm) under consideration in this paper. We note that in recent experiments pulse THz lasers with strengths >MV/cm have been achieved and interband high-order harmonic generation has been observed under such strong THz fields^25^. Such strong fields would definitely induce interesting non-adiabatic geometric phase effects, which, however, are not the topic of this paper.

The excitation by a weak linearly polarized optical laser is described by the interaction Hamiltonian 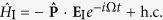 Here 
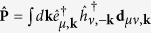
 is the interband polarization operator, where 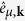 (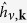) annihilates an electron (hole) with momentum **k** and spin or valley index *μ* (*ν*). The interband dipole moment **d**_*μv*, **k**_ is^16^,^26^
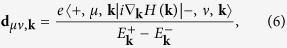
where + and – denote the conduction and valence bands, respectively, 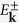 the band energy, and 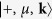
(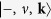) is the Bloch state of the conduction (valence) band with momentum **k** and spin or valley index *μ* (*ν*). If we assume that the semiconductor is initially in the “vacuum” state 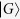 with empty conduction bands and filled valence bands, the linear optical response is^20^
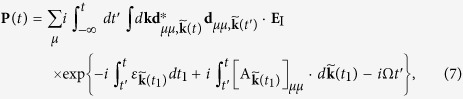
where 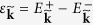 is the energy of the electron-hole pair and 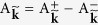 is the combined Berry connection of the electron-hole pair. Then the *N*th sideband emission (at frequency Ω + 2*Nω*) is
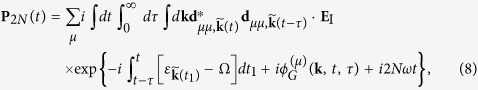
where 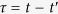 denotes the evolution time between the excitation and the emission, and
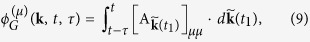
is the geometric phase. Here for the sake of simplicity we have assumed that the Berry connection is Abelian (i.e., the THz field does not mix near-degenerate bands of different spin or valley indices). The generalization to non-Abelian case is possible^20^.

We should remark on the approximations used in derivation of equations (7) and (8). First we use the single-particle approximation. This is justified since we consider weak optical excitation (in contrast to the strong THz field) and assume only single electron-hole pairs are excited. Second, in the single-particle dynamics, we neglect effects such as electron scattering, thermal diffusion, and phonon scattering. By comparing the theoretical computation with experimental results^19^, we found that these effects can be captured essentially by including a phenomenological relaxation term *e*^−*γτ*^ in equations (7) and (8). Finally for the sake of simplicity, we do not consider Coulomb interaction between the electron and the hole and hence neglect the excitonic effects. This will be justified later when we consider HSG in monolayer MoS_2_ (see the section of numerical results). In sum, the formulas (7) and (8) with the electron-hole pair relaxation, is conceptually simple, whereas can capture the essential physics.

Generally, the geometric phase is much smaller than the dynamical phase accumulated during a cyclic evolution under the THz field driving (since the kinetic energy of electron-hole pairs is much greater than the energy correction due to interband coupling induced by the THz field). Hence the geometric diffusion induced by the geometric phases is also much smaller than the dynamical diffusion and may be neglected. However, we shall demonstrate that, in configurations where the dynamical diffusion terms cancel each other, the imaginary geometric phases have observable effects on the sideband emission in time-reversal symmetric materials with non-vanishing Berry curvatures. Essentially, the geometric and dynamical phases can be separated by their different behaviours under the time-reversal (TR) transformation.The wavepacket can be initially prepared in a superposition of a pair of TR related states 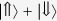 (by optical excitation, e.g., as in the example discussed later in this paper). Then these two states obtain the same dynamical phase *ϕ*_*D*_ during the adiabatic evolution because they have the same eigenenergy. However, 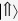 and 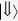 get the opposite geometric phases ±*ϕ*_*G*_ since the Berry connection reverses sign under TR transformation [see^13^ or equation (11)]. Thus the superposition state becomes 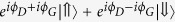 and hence *ϕ*_*D*_ becomes a global phase. In next section we will show that by interference between time-reversal related quantum trajectories, we can observe the real part of the geometric phase as an FR of the sideband emission^20^,^21^, and the imaginary part as the PE of the sidebands.

### Faraday rotation and polarization ellipticity of THz sidebands

We take monolayer MoS_2_ as a model system. This material has two time-reversal related valleys ±*K* at the corners of the 2D hexagonal Brillouin zone [Fig. 2(b)], where the strong spin-orbit coupling causes a spin splitting of about 160 meV at the valence band top^27^. We assume that the optical laser is near-resonant with the transitions between the band edges of the conduction band and the highest valence band, and therefore neglect the transitions from the lower valence bands. The energy bands near the band edge can be effectively described by the Hamiltonian^27^
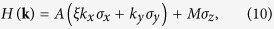
where *A* = 3.51 eV·Å, the band gap 2*M* = 1.9 eV, *ξ* = ±1 denotes the ±*K* valley, and **k** is measured from the respective Dirac points at valleys ±*K*^27^,^28^,^29^. The energy spectrum is 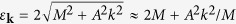 with two-fold valley degeneracy. The Berry connection and dipole moment at ±*K* valleys (labelled by the pseudo-spin 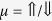) satisfy the time-reversal relations
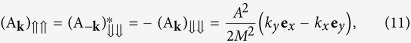

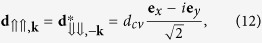
where 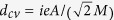. Note that the optical selection rules are such that the interband transition at valley +*K* (–*K*) is coupled exclusively with the *σ*^+^ (*σ*^–^) polarized light.

The effects of the geometric phases on the HSG in monolayer MoS_2_ or similar materials can be intuitively understood. A laser with linear polarization *σ*^+^ + *σ*^−^ causes equal transitions in +*K* and −*K* valleys at time *t*′ and creates an electron-hole pair in the superposition state 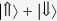. After driving by the THz field, the quantum trajectories at the two valleys obtain the same dynamical phase 
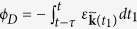
 but opposite geometric phases 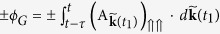 [see equation (8) and Fig. 2(b)], so the superposition state becomes 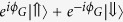. Thus the emission by the recombination of the electron-hole pair at time *t* has polarization *e*^*iϕ_G_*^*σ*^+^+*e*^*−iϕ_G_*^*σ*^−^. The real part of *ϕ*_*G*_ induces a phase shift between the two circular polarizations and hence an FR ℜ*ϕ*_*G*_ 20,21, while the imaginary part induces an amplitude difference between the two circular polarizations and hence a PE ℑ*ϕ*_*G*_ (assumed <<1) [Fig. 2(a)].

**Figure 3:**
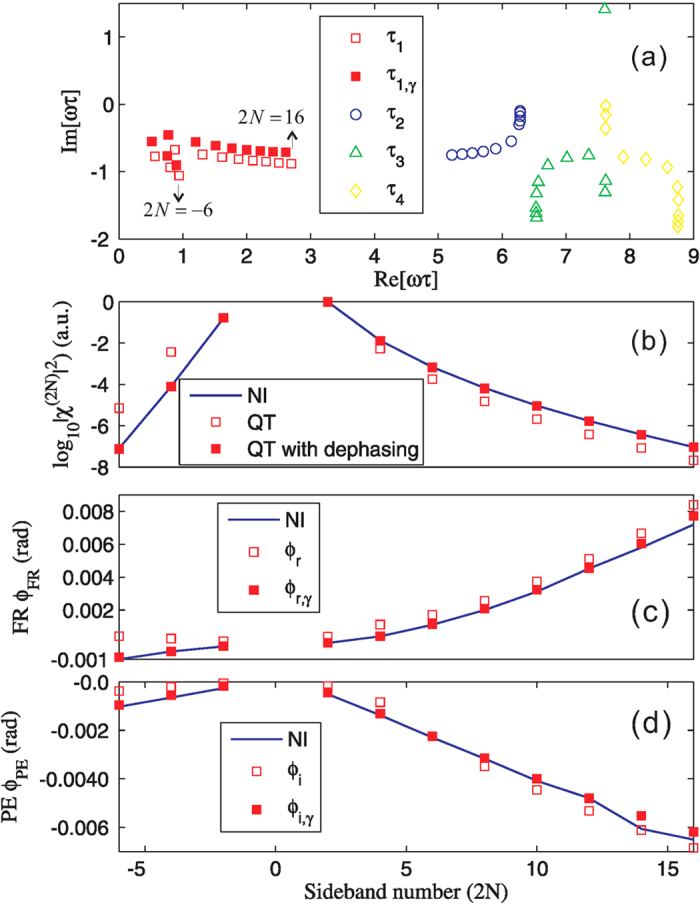
Faraday rotation and polarization ellipticity of THz sidebands in monolayer MoS_2_. (**a**) shows the stationary phase points *τ*_*n*_ for 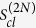 with the sideband order 2*N* = −6 → 16. Only the first four stationary phase points (*n* = 1 → 4) are given for each sideband. The filled squares are the first (*n* = 1) stationary phase points *τ*_1,*γ*_ that includes the dephasing effect [solutions of equation (27)]. (**b**) shows the relative intensities of the sidebands, while (**c**) and (**d**) give the corresponding FR and PE of them. In (**b**-**d**), the lines are obtained by numerical integration of equation (13), and the open and filled squares show the quantum trajectory (QT) results obtained using the dominant stationary phase points (*t*_1_, *τ*_1_) without dephasing effect [the first physical solution to equation (26)] and (*t*_1,*γ*_, *τ*_1_,_*γ*_) with the dephasing effect [the first physical solution to equation (27)], respectively.

Now we insert 
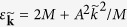
 and equations (11, 12) into equation (8). By taking 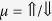, we obtain the linear susceptibility for the *σ*^+^/*σ*^−^-polarized optical field **E**_I_. After a direct computation, the relative amplitude of the *N*th sideband for the *σ*^±^-polarized optical field is obtained as
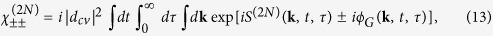
where the geometric phase is obtained from equation (9)
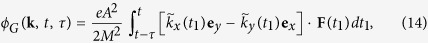
and the action is
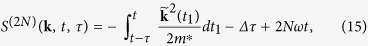
with m^*^ = *M*/(2*A*^2^) being the reduced effective mass of the electron-hole pair and Δ = 2*M* − Ω the optical laser detuning. Since *ϕ*_*G*_/*S*^(2*N*)^ ∼ *ω*/(2*M*) << 1, i.e., the geometric phase is much less than the dynamical phase, the quantum trajectories are determined by the stationary phase conditions for the dynamical phase, i.e.,
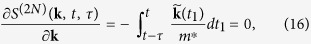

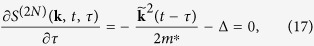

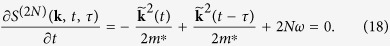
Note that 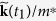 is the semiclassical velocity of the electron-hole pair. Hence equation (16) means the electron accelerated by the THz field returns to the hole after *τ* for recombination. Equations (17) and (18) express the energy conservation conditions for the excitation of the electron-hole pair at time *t* − *τ* and for the sideband generation through electron-hole pair recombination at time *t*, respectively. By equation (14), the geometric phase accumulated along the quantum trajectory determined by equations (16)-(18), 43, 44 is
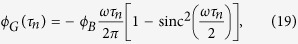
with 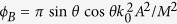 being the Berry phase an electron-hole pair acquires in a full THz period, and (**k**_*cl*_, *t*_*n*_, *τ*_*n*_) being solution of equations (16)-(18), 43, 44. Generally, the solutions to equations (16)-(18), 43, 44 are not real numbers, i.e., *τ*_*n*_ is a complex number [Fig. 3(a)]. This may happen when the detuning Δ > 0 [c.f. equation (17)], where the energy conservation cannot be satisfied by real solutions (virtual processes are involved). On the other hand, for high sidebands with 2*Nω* > 3.17|cos(2*θ*)|*U*_*p*_, where *U*_*p*_ = *e*^2^*F*^2^/(4m^*^ω^2^) is the ponderomotive energy and 3.17|cos(2*θ*)|*U*_*p*_ is the cut-off frequency for HSG^17^,^23^, the electron-hole pair cannot gain sufficiently large kinetic energy along real trajectories, and we can only have complex solutions. Finally, even if we have Δ < 0 and 2*Nω*≤3.17|cos(2*θ*)|*U*_*p*_, complex solutions exist if the polarization ellipticity *θ* of the THz field is big enough. Thus *τ*_*n*_ determines a complex quantum trajectory and leads to an imaginary part of the geometric phase *ϕ*_*G*_(*τ*_*n*_). In particular, if one of the stationary phase points dominates, say (**k**_*cl*_, *t*_*1*_, *τ*_*1*_), we have 
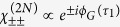
. If the optical laser has a linear polarization 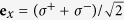, the polarization of the *N*th sideband is
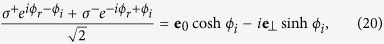
where 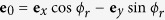 and 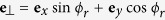 with *ϕ*_*r*_ and *ϕ*_*i*_ being the real and imaginary parts of the geometric phase *ϕ*_*G*_(*τ*_1_), respectively. That gives the FR *ϕ*_*r*_ and PE *ϕ*_*i*_ (for *ϕ*_*i*_ << 1).

### Numerical results

In order to confirm the validity of the quantum trajectory method, we calculate the susceptibilities by direct numerical integration of equation (13), and compare the FR and PE of the sidebands with the real and imaginary parts of the geometric phases at the stationary phase points. In the calculation, the THz field is set such that *ω* = 2 meV, *F*_THz_ = 10 kV/cm and *θ* = *π*/6. We take a CW optical laser that is tuned below the band gap (2*M* = 1.9 eV) by Δ = 2*ω*, and we assume its strength is weak enough. To describe the scattering effect in real materials, we include a phenomenological dephasing term of the electron-hole pair e^−*γτ*^ in the integration of equation (13), with *γ* = 3 meV. Such dephasing can be due to phonon scattering, relaxation of the electron-hole pair to bound exciton states and so on.

For the sake of simplicity, we neglect the Coulomb interaction between the electron and hole, which is justified for the following reasons. First, the exciton binding energy (100s of meV^30^) is much larger than *ω* and Δ and hence the exciton bound states are far off-resonant from the optical excitation. Also the exciton emission would be at frequencies 100s of meV below Ω, which is well separated from the THz sideband frequencies. Thus the relaxation of the electron-hole pair to bound exciton states would only reduce the overall intensity of the sideband emission, but leave the polarization of the sideband unaffected. In fact, the free particle approximation has been verified by comparing the theoretical computation with the experiment results^19^. The relaxation of electron-hole pairs to the bound exciton state may also affect the HSG. However, the relaxation time of an electron-hole pair to an exciton is a few picoseconds^31^, which is long enough for the electron-hole pair to complete the quantum trajectories to emit the sideband photons [see Fig. 3(a), *τ*_1_ = 1 ∼ 3*T*, where *T* ~ 2 ps is the period of the THz field].

The comparison is shown in Fig. 3. The results of the sideband strength in Fig. 3b suggest that the electron-hole pair evolution is well approximated by the dominant trajectory with *n* = 1. The numerically calculated FR and PE of the sidebands are almost equal to the real and imaginary parts of the geometric phases *ϕ*_*G*_ accumulated along the first quantum trajectory [determined by the first physical solution *τ*_1_ of equations (16)-(18), 43, 44], respectively. The Faraday rotation and polarization ellipticity are 0.001 ~ 0.01 rad, which can be readily detected experimentally.

## Discussion

In Fig. 3, the discrepancy between the quantum trajectory and the numerical integration results is due to the contribution from other trajectories and the changing of the stationary phase points by the inclusion of the dephasing term in the action,
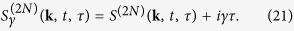
The modified solutions (**k**_*cl*_, *t*_*n,γ*_, *τ*_*n*_) satisfy the corresponding stationary phase conditions for 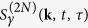. *τ*_1,*γ*_ and their corresponding geometric phases *ϕ*_*r*/*i,γ*_ are also shown in Fig. 3, which indeed agree better with the numerical results.

We observe in Fig. 3 that both the FR and the PE increases almost linearly with the sideband order 2*N* > 0. This can be understood as follows. The electron-hole pair need to go along a longer trajectory [i.e. larger *τ* as shown in Fig. 3(a)] in the **k**-space to acquire a higher kinetic energy 2*Nω* − Δ, which in turn leads to a larger geometric phase of the wavepacket [see equation (19)].

## Method

### Quantum trajectory method

By solving equation (16), we obtain the stationary momentum **k**_*cl*_ as
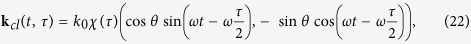
where *χ*(*τ*) = sinc(*ωτ*/2). Under the stationary phase approximation for 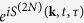 (which approximates the electron-hole evolution by quantum trajectories plus quantum fluctuations around them), equation (13) can be integrated as
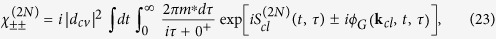
where the semiclassical action is
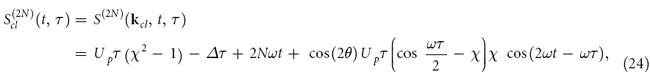
with *U*_*p*_ = *e*^ 2^*F*^ 2^/(4m^*^ω^ 2^) being the ponderomotive energy. Using the stationary phase approximation again for *t* and *τ*, equation (23) becomes^32^,^33^
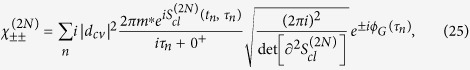
where the stationary phase point (*t*_*n*,_*τ*_*n*_) satisfies
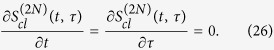
We solve equation (26) numerically as shown in Fig. 3(a) and then obtain the optical susceptibilities of the *N*th sideband using equation (25). If the dephasing term is included, the modified stationary phase points (*t*_n,*γ*_, *τ*_n,*γ*_) satisfy
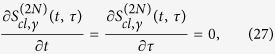
where 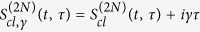.

### Numerical integration method

The integration in equation (13) over **k** can be calculated analytically:
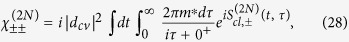
where
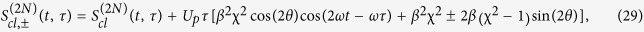
with 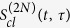 given in equation (24) and *β* = *ω*/(4*M*). Using the properties of Bessel function, equation (28) can be written as
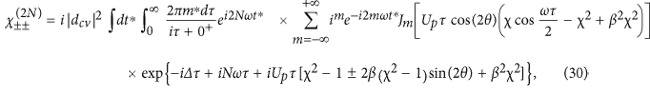
where 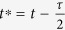. Then we get the integration in equation (30) over *t* (i.e. the Fourier transform) as
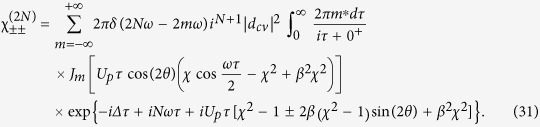
We apply the standard numerical integration method to equation (31) and then obtain the optical susceptibilities of the *N*th sideband.

## Additional Information

**How to cite this article**: Yang, F. and Liu, R.-B. Geometric diffusion of quantum trajectories. *Sci. Rep.* **5**, 12109; doi: 10.1038/srep12109 (2015).
